# Assessment of high-light adaptation and screening of superior oat germplasm

**DOI:** 10.3389/fpls.2026.1798891

**Published:** 2026-04-10

**Authors:** Bohong Zhang, Xiang Ma, Zeliang Ju, Jingjie Liu, Zhifeng Jia

**Affiliations:** 1 Qinghai Provincial Key Laboratory of Adaptive Management on Alpine Grassland, Qinghai University, Xining, China; 2 Key Laboratory of Development of Forage Germplasm in the Qinghai-Tibetan Plateau of Qinghai Province, Academy of Animal Science and Veterinary, Qinghai University, Xining, China

**Keywords:** adaptability, comprehensive evaluation, high-light stress, oat, structural equation modeling

## Abstract

**Background:**

In the context of climate warming, high-light stress has become a major environmental constraint on crop photosynthetic efficiency and growth. Elucidating the physiological and morphological responses of oat seedlings to high light, together with the identification of tolerant germplasm, is therefore essential for enhancing oat adaptation to light-intensive environments.

**Methods:**

Forty-eight oat (*Avena sativa*) accessions were assessed for high-light adaptability at the seedling stage. A preliminary light-gradient experiment using four representative accessions identified 1600 μmol·m^-2^·s^-1^ as an optimal light intensity for high-light stress screening. All accessions were subsequently exposed to this light regime, and nine traits were quantified, including plant height, leaf thickness, stem diameter, aboveground fresh and dry weights, SPAD value, net photosynthetic rate, proline (Pro) content, and ribulose-1,5-bisphosphate carboxylase/oxygenase (Rubisco) activity. Correlation analysis, cluster analysis, the TOPSIS multi-criteria decision model, and structural equation modeling were integrated to comprehensively evaluate high-light adaptability.

**Results:**

High-light stress markedly suppressed seedling growth, with most growth-related traits exhibiting an overall decline. Based on the comprehensive evaluation index derived from the TOPSIS model in combination with cluster analysis, the 48 oat accessions were classified into four distinct high-light tolerance groups. Among these, Qingyongjiu 478 and Xizangbailang demonstrated superior adaptation to high-light conditions. Structural equation modeling further indicated that proline content, SPAD value, and stem diameter had significant effects on aboveground fresh weight formation, with total effects of −0.506, 0.475, and 0.470, respectively.

**Conclusion:**

This study establishes a robust framework for evaluating high-light adaptability at the oat seedling stage, identifies key physiological traits governing biomass accumulation under high-light stress, and provides valuable germplasm resources and theoretical support for high-light tolerance gene discovery and oat breeding programs.

## 1 Introduction

The Qinghai-Tibetan Plateau and other alpine pastoral regions are characterized by cold and arid climates, featuring prolonged cold seasons and brief warm periods (Li et al., 2020). Natural grasslands in these environments exhibit inherently low primary productivity and a severely constrained growing season (Wang et al., 2022), leading to a persistent scarcity of forage resources available for green harvesting during winter and spring and a pronounced imbalance between forage supply and livestock demand. In recent decades, under the combined influences of global climate warming, the increasing frequency of extreme climatic events, overgrazing, and suboptimal grassland management practices, grassland degradation in alpine regions has intensified. As a consequence, grassland ecosystem stability has declined, forage yields have become increasingly insufficient, and seasonal disparities in forage availability have been exacerbated, collectively imposing substantial constraints on the sustainable development of pastoral livestock systems (Zhou et al., 2023). Under these circumstances, the expansion of cultivated forage systems has become a key strategy for stabilizing forage supply and alleviating pressure on natural grasslands. However, in alpine environments, forage crop productivity is largely governed by their ability to withstand and adapt to multiple abiotic stresses, underscoring the critical importance of enhancing stress tolerance in forage species cultivated in high-altitude regions. In alpine regions, geographic and atmospheric conditions give rise to exceptionally intense solar radiation during the summer growing season (Liu B. et al., 2025). Under clear-sky conditions, direct irradiance incident on leaf surfaces can reach 60 000~100 000 lx (approximately 1 080~1 800 μmol·m^-2^·s^-1^), substantially exceeding the light saturation point of most crops (Wang et al., 2020). Such high irradiance is frequently accompanied by elevated land surface temperatures and enhanced soil moisture evaporation, thereby exacerbating concurrent heat and drought stresses. Under these combined conditions, crops commonly experience declines in photosynthetic efficiency and limitations in biomass accumulation (Sharma et al., 2023), rendering high-light stress an increasingly critical environmental constraint on the normal functioning of the photosynthetic apparatus in forage crops cultivated in alpine ecosystems. Consequently, the identification of forage cultivars with strong high-light tolerance and ecological adaptability is essential for ensuring stable forage supply, alleviating forage–livestock imbalances, and supporting the sustainable development of animal husbandry in these regions. Oat (*Avena sativa*), a dual-purpose cereal and forage grass species, is characterized by cold tolerance, drought resistance, adaptability to low-fertility soils, and broad ecological plasticity (Wu et al., 2025), making it a key species for artificial grassland establishment and winter-spring forage supplementation in alpine and agro-pastoral transition zones.

Light serves as the primary energy source for photosynthesis and is a fundamental regulatory factor governing plant growth and development, playing a pivotal role in circadian regulation and environmental signal integration (Li et al., 2022). Optimal light conditions support the stable functioning of the photosynthetic apparatus, enhance light-use efficiency, and ultimately promote biomass accumulation and yield formation. However, when incident light intensity exceeds the photosynthetic capacity of plants or fluctuates sharply, excess excitation energy cannot be effectively dissipated through photochemical reactions, thermal dissipation, or chlorophyll fluorescence (Bassi and Dall’Osto, 2021). This disruption of the dynamic balance between light energy absorption and utilization leads to the overproduction of reactive oxygen species (ROS), resulting in oxidative damage to the photosynthetic electron transport system. Among the components of the photosynthetic machinery, photosystem II (PSII) is the central hub for light energy absorption, transfer, and dissipation, and is therefore particularly susceptible to photodamage under high-light conditions (Gilmore and Govindjee, 1999). Damage to PSII inevitably reduces photosynthetic efficiency and constrains biomass production. To prevent or alleviate photoinhibition, photosynthetic organisms have evolved a suite of multilayered photoprotective and regulatory mechanisms during long-term evolution. These include the dissipation of excess excitation energy via non-photochemical quenching (Derks et al., 2015), regulation of electron transport pathways such as cyclic electron flow (Yamori and Shikanai, 2016), enhancement of antioxidant defense systems (Asada, 2006), maintenance of PSII repair processes (Mulo et al., 2008), and avoidance strategies involving leaf and chloroplast movements. These photoprotective strategies vary among species and genotypes, which is reflected in marked variation in adaptation to high irradiance and light-use efficiency. Therefore, systematic identification and comprehensive evaluation of high-light tolerance in oat germplasm are of great significance for screening elite genetic resources capable of efficient light utilization under strong irradiance. In recent years, studies on stress tolerance in oat germplasm have primarily focused on traits such as salinity-alkalinity tolerance (Peng et al., 2025), cold resistance, drought tolerance (Sadras et al., 2017), lodging resistance (Liu et al., 2024), and pest resistance. These investigations have substantially advanced our understanding of the physiological responses and adaptive mechanisms of oats to diverse abiotic stresses, providing an important foundation for elite germplasm identification and genetic improvement. Nevertheless, most existing studies have addressed the impacts of abiotic stresses on oat photosynthesis from isolated or indirect perspectives, and systematic assessments of photosynthetic response characteristics and high-light adaptive capacity among diverse oat germplasm under high-light stress conditions remain largely lacking.

Accordingly, this study employed 48 high-yielding and high-quality oat germplasm accessions screened from previous field trials by our research group to conduct high-light stress experiments at the seedling stage. A suite of nine morphological, physiological, and photosynthetic traits was systematically assessed, including plant height, leaf thickness, stem diameter, aboveground fresh and dry biomass, SPAD value, net photosynthetic rate, Pro content, and Rubisco activity. High-light tolerance coefficients were calculated, and a combination of analysis of variance, correlation analysis, cluster analysis, and the TOPSIS multi-criteria decision-making approach was applied to comprehensively evaluate the adaptive performance of oat germplasm under high-light conditions. The overarching objective was to identify elite accessions exhibiting superior high-light tolerance, thereby providing both a theoretical foundation and parental resources for the breeding of high-light-tolerant oat cultivars. Furthermore, structural equation modeling was used to disentangle the regulatory pathways through which genotype, light intensity, and their interactions influence aboveground fresh biomass accumulation. By partitioning directxand indirect effects, this analysis aimed to identify the key traits governing biomass formation under high-light stress and to elucidate their functional pathways across contrasting oat genotypes.

## 2 Materials and methods

### 2.1 Plant materials

A total of 48 oat germplasm accessions were evaluated in this study. The accessions originated from 16 countries and were provided by the Grassland Research Institute, Academy of Animal and Veterinary Sciences, Qinghai University. Accessions from Europe constituted the majority of the collection (68.75%). Detailed information on accession names and geographic origins is presented in **Table 1**.

**Table 1:** Names and sources of the tested germplasm resources.

Code	Germplasm resources	Origin	Code	Germplasm resources	Origin
V1	Qingyongjiu 471	Canada	V25	Qingyongjiu 023	Soviet Union
V2	Qingyongjiu 465	Sweden	V26	Qingyongjiu 027	Soviet Union
V3	Qingyongjiu 458	Sweden	V27	Qingyongjiu 062	Romania
V4	Qingyongjiu 488	France	V28	Qingyongjiu 064	Soviet Union
V5	Qingyongjiu 085	Switzerland	V29	Qingyongjiu 097	Sweden
V6	Qingyongjiu 084	Germany	V30	Qingyongjiu 160	Denmark
V7	Zhangyan 1	China	V31	Qingyongjiu 479	Romania
V8	Xizangbailang	China	V32	Qingyongjiu 480	Victoria
V9	Qingyongjiu 045	Canada	V33	Qingyongjiu 486	Sweden
V10	Qingyongjiu 051	Germany	V34	Qingyongjiu 482	Australia
V11	Qingyongjiu 080	Hungary	V35	Qingyongjiu 092	Hungary
V12	Qingyongjiu 083	Germany	V36	Qingyongjiu 090	Switzerland
V13	Qingyongjiu 257	Denmark	V37	Qingyongjiu 061	Romania
V14	Qingyongjiu 326	Denmark	V38	Qingyongjiu 060	Bulgaria
V15	Qingyongjiu 476	Japan	V39	Qingyan 5	China
V16	Qingyongjiu 475	Netherlands	V40	Menggu Oat	China
V17	Qingyongjiu 477	Japan	V41	Bayan 5	China
V18	Qingyongjiu 478	Japan	V42	Gaohan 7	China
V19	Qingyongjiu 226	Denmark	V43	Qingyongjiu 052	Hungary
V20	Qingyongjiu 178	Denmark	V44	Qingyongjiu 055	Romania
V21	Qingyongjiu 073	Hungary	V45	Qingyongjiu 057	Mongolia
V22	Qingyongjiu 066	Hungary	V46	Qingyongjiu 059	Bulgaria
V23	Qingyongjiu 037	Canada	V47	Qingyongjiu 489	France
V24	Qingyongjiu 029	Canada	V48	Qingyongjiu 089	Soviet Union

### 2.2 Experimental design

#### 2.2.1 Determination of an appropriate high-light stress intensity

The experiment was conducted in March 2025 in a walk-in growth chamber at the Academy of Animal and Veterinary Sciences, Qinghai University. Environmental conditions were maintained at a day/night temperature of 25/20 °C, relative humidity of 55%, a basal light intensity of 400 μmol·m^-2^·^-1^, and a 14 h light/10 h dark photoperiod. Four oat accessions were randomly selected from a total of 48 germplasm resources for a preliminary experiment. Uniform, well-filled seeds were surface-sterilized in 1% sodium hypochlorite for 10 min, rinsed thoroughly with distilled water, and evenly placed in square germination boxes (11 cm × 11 cm × 3 cm) lined with filter paper, with 30 seeds per box. After 7 d of germination, seedlings exhibiting uniform growth and vigorous development were transplanted into plastic pots (13.5 cm in diameter and 13.5 cm in height), with four seedlings per pot. Plants were grown in a commercial general-purpose substrate (Yangfeng, China) consisting mainly of peat, coco coir, perlite, and vermiculite, with an organic matter content ≥ 40% and total nutrients [N+P_2_O_5_+K_2_O] ≥ 1%. During the seedling stage, plants were irrigated every 7 d to maintain normal growth. High-light stress treatments were initiated when seedlings reached the three-leaf stage. Three high-light levels (1200, 1600, and 2000 μmol·m^-2^·^-1^) were applied using a manufacturer-customized high-intensity lighting panel to ensure uniform illumination, with 400 μmol·m^-2^·^-1^ serving as the control (CK) (Uraguchi et al., 2009). The daily light period extended from 06:00 to 20:00, during which high-light stress was imposed between 10:00 and 17:00, while control light intensity was maintained during the remaining hours. All treatments were applied for 14 d. For each accession and light treatment, three pots were used as biological replicates, and one uniformly growing plant per pot was selected for subsequent morphological and physiological measurements.

#### 2.2.2 Evaluation of high-light adaptability

Based on the results of the preliminary experiment, 1600 μmol·m^-2^·^-1^ was selected as the high-light stress intensity, with 400 μmol·m^-2^·^-1^ used as the control. High-light adaptability of 48 core oat germplasm accessions was evaluated at the seedling stage. Cultivation conditions and the timing of high-light exposure were identical to those used in the preliminary experiment.

### 2.3 Trait measurements

#### 2.3.1 Agronomic traits

After 14 d of treatment, agronomic traits were measured for the selected plants. Plant height was determined using a steel measuring tape (accuracy: 0.1 cm). Leaf thickness and stem diameter were measured with a digital vernier caliper (accuracy: 0.01 mm). Aboveground fresh weight was determined using an electronic balance (accuracy: 0.1 mg). Samples were then heated at 105 °C for 30 min to deactivate enzymes, followed by oven-drying at 65 °C to constant weight to determine dry biomass.

#### 2.3.2 SPAD value and net photosynthetic rate

The SPAD value of the flag leaf was measured using a SPAD-502 portable chlorophyll meter (Konica Minolta, Japan). Measurements were taken at the basal, middle, and upper sections of the flag leaf, and the mean of three readings was recorded as the SPAD value for each plant. Net photosynthetic rate (A) was measured between 15:00 and 16:30 on the 14th day of treatment using a LI-6800 portable photosynthesis system (LI-COR, USA) within the growth chamber. Light intensity was set at 400 μmol·m^-2^·^-1^ for the control and 1600 μmol·m^-2^·^-1^ for the high-light treatment. Measurements were conducted under a red–blue light source with an open gas-exchange system. Data were recorded after signal stabilization, with three measurements per plant, and averaged for subsequent analysis.

#### 2.3.3 Proline content and photosynthetic enzyme activity

After 14 d of treatment, leaf samples were harvested, immediately frozen in liquid nitrogen, and stored at -80 °C for physiological analyses. Pro content and Rubisco activity were quantified using commercial assay kits (Suzhou Comin Biotechnology Co., Ltd., China), following the manufacturer’s protocols.

#### 2.3.4 Statistical analysis

Mean values from three biological replicates for each trait were used to calculate the high-light tolerance coefficient, defined as the ratio of the mean value under high-light stress to that under control conditions. These coefficients were subsequently used for TOPSIS-based comprehensive evaluation and cluster analysis. Data processing and preliminary statistical analyses were performed using Microsoft Excel 2019, whereas multivariate and diversity analyses were conducted in SPSS 27.0. Pearson correlation analysis and Mantel tests were performed in R version 4.4.2 using the dplyr, linkET, and ggplot2 packages. The TOPSIS multi-criteria decision-making model was implemented with data organization and weighted calculations conducted using the plyr package, and objective weights of evaluation indices were determined using the entropy weight method. Key trait selection was performed using the randomforest package. Piecewise structural equation modeling was conducted using the PiecewiseSEM package. Standardized effect sizes and cluster diagrams were generated using Origin 2024, while all other figures were produced with GraphPad Prism 9.

## 3 Results

### 3.1 Determination of an appropriate light intensity for high-light tolerance evaluation

**Figure 1:**
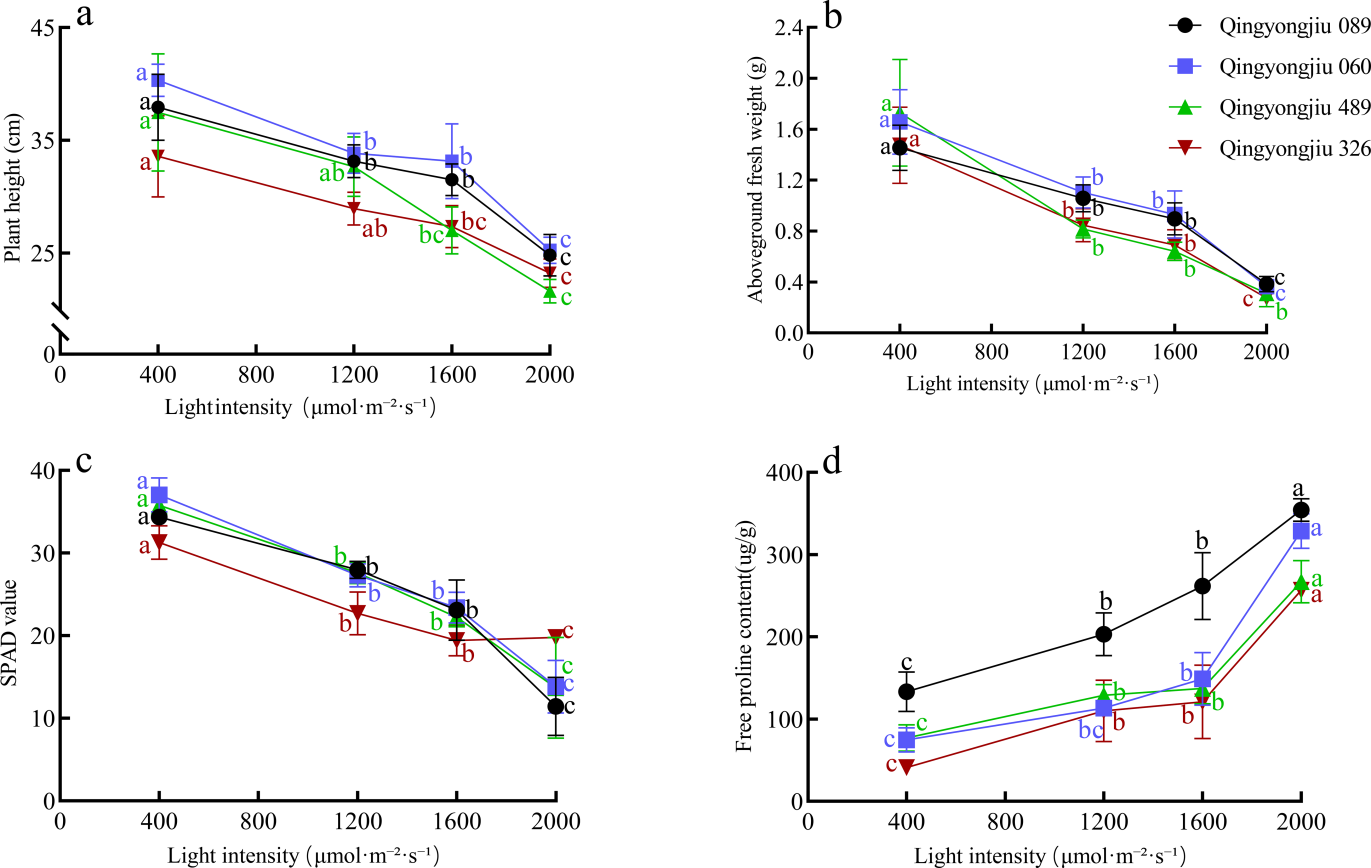
Changes in **(a)** plant height, **(b)** aboveground fresh weight, **(c)** SPAD value, and **(d)** free proline content in four oat germplasms under light intensities of 400, 1200, 1600, and 2000 μmol·m⁻²·s⁻¹. Different letters indicate significant differences among treatments within the same genotype (*P* < 0.05).

To establish an appropriate light intensity for assessing high-light tolerance in oat, four genotypes were randomly selected from a collection of 48 accessions and subjected to a gradient of light intensity treatments (**Figure 1**). As light intensity increased, plant height, aboveground fresh weight, and SPAD values consistently declined across all genotypes, whereas proline content exhibited a progressive increase. Under a light intensity of 1200 μmol·m^-2^·^-1^, changes in plant height and proline content were not significant for some genotypes relative to the control, and overall phenotypic and physiological responses were relatively limited, hampering effective discrimination of genotypic differences in high-light tolerance. In contrast, exposure to 2000 μmol·m^-2^·^-1^ resulted in a pronounced reduction in plant height, aboveground biomass, and SPAD values in all genotypes; seedling growth was severely suppressed, leaves displayed evident wilting, and aboveground fresh weight tended to converge among genotypes, thereby obscuring tolerance-related differences. Notably, at 1600 μmol·m^-2^·^-1^, all measured traits, including plant height, aboveground biomass, SPAD value, and proline content, differed significantly from those of the control (*P* < 0.05), allowing clear and stable differentiation of high-light tolerance among genotypes. Collectively, these results indicate that 1600 μmol·m^-2^·^-1^ represents an optimal light intensity for high-light tolerance evaluation in oat.

### 3.2 Effects of high-light stress on seedling traits in oat

**Figure 2:**
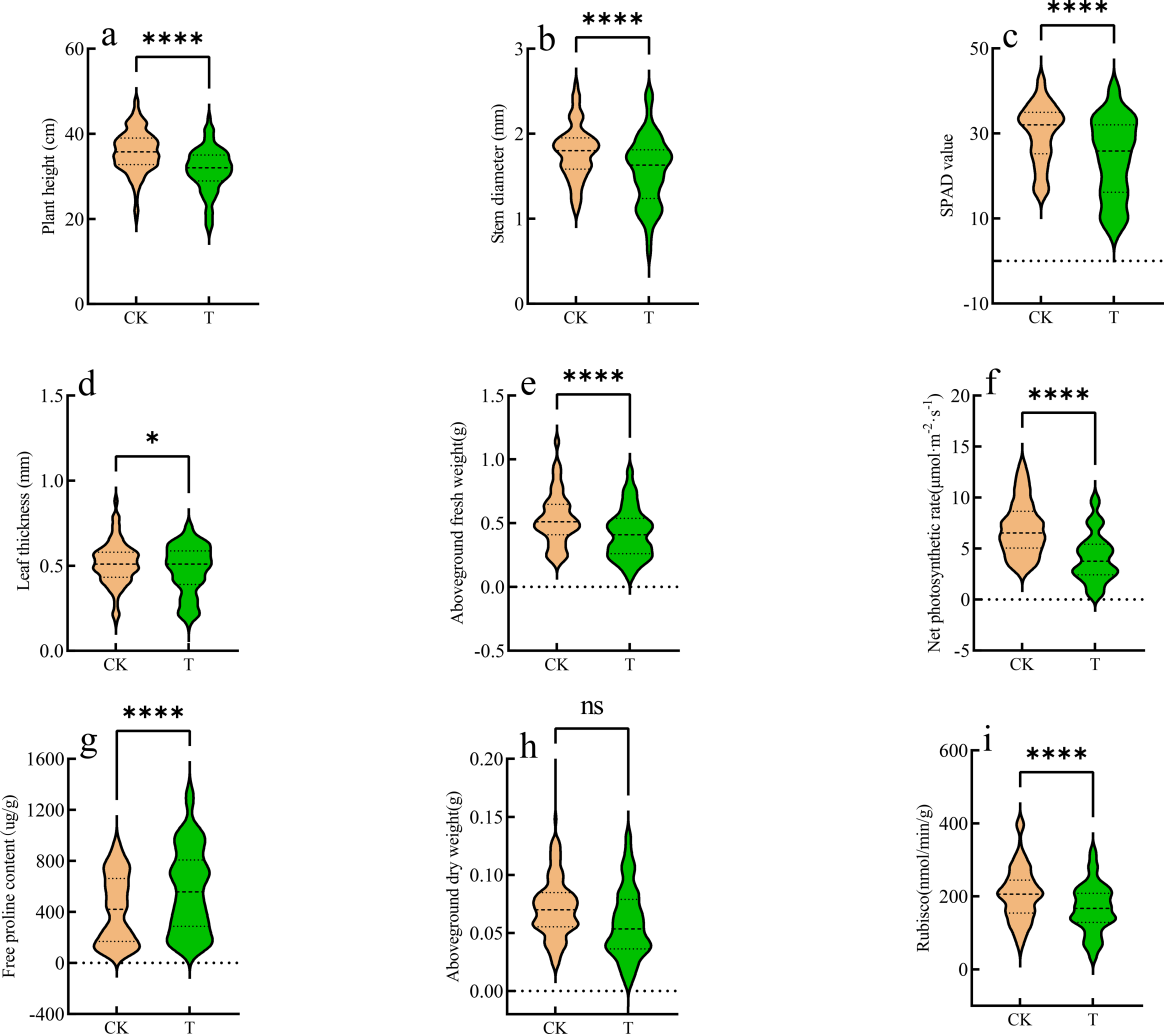
Violin plots showing the distribution of **(a)** plant height, **(b)** stem diameter, **(c)** SPAD value, **(d)** leaf thickness, **(e)** aboveground fresh weight, **(f)** net photosynthetic rate, **(g)** free proline content, **(h)** aboveground dry weight, and **(i)** Rubisco activity among 48 oat germplasms under CK and T conditions. *P < 0.05; ****P < 0.0001; ns, not significant at the 0.05 level.

Under a light intensity of 1600 μmol·m^-2^·^-1^, the responses of 48 oat genotypes at the seedling stage are presented in **Table 2**; **Figure 2**. Compared with the control, high-light stress significantly decreased plant height, stem diameter, SPAD value, leaf thickness, aboveground fresh weight, net photosynthetic rate, and Rubisco activity (*P* < 0.05), while proline content increased significantly (*P* < 0.05), indicating that high-light conditions markedly inhibited both growth and photosynthetic physiology (**Figures 2a-g, i**). Among the measured traits, net photosynthetic rate exhibited the largest decline, with a mean of 4.13 μmol·m^-2^·^-1^, corresponding to a 40.83% reduction relative to the control (**Figure 2f**), reflecting substantial suppression of carbon assimilation. In contrast, aboveground dry weight did not differ significantly from the control (*P*>0.05), suggesting that short-term high-light stress exerted a limited effect on dry matter accumulation (**Figure 2h**).

**Table 2:** Descriptive statistics of traits in 48 oat resources under control and high-light stress conditions.

Indicator	Control treatment			High light stress treatment		
	Range	Mean ± standard deviation	Coefficient of variation (CV, %)	Range	Mean ± standard deviation	Coefficient of variation (CV, %)
Plant Height (cm)	20.10~47.80	35.82 ± 4.95	13.82	17.00~44.00	31.51 ± 5.29	16.79
Stem Diameter (mm)	1.08~2.59	1.81 ± 0.30	16.57	0.58~2.61	1.55 ± 0.40	25.81
SPAD value	14.70~43.50	30.19 ± 6.98	23.12	5.00~42.20	24.37 ± 9.09	37.30
Leaf Thickness (mm)	0.21~0.87	0.52 ± 0.11	21.15	0.15~0.74	0.46 ± 0.14	30.43
Aboveground Fresh Weight (g)	0.18~1.15	0.54 ± 0.21	38.89	0.07~0.96	0.42 ± 0.20	47.62
Aboveground Dry Weight (g)	0.02~0.15	0.07 ± 0.03	42.86	0.01~0.14	0.06 ± 0.03	50.00
Net Photosynthetic Rate (μmol·m^-2^·s^-1^)	2.55~13.55	6.98 ± 2.63	37.68	0.84~10.19	4.13 ± 2.37	57.38
Free Proline Content (μg/g)	68.39~975.78	422.41 ± 266.55	63.10	102.18~1357.09	571.95 ± 328.76	57.48
Rubisco(nmol/min/g)	64.30~411.52	210.43 ± 69.05	32.81	25.72~334.36	168.22 ± 66.95	39.80

Coefficient of variation (CV) analysis (**Table 2**) indicated that all traits exhibited varying degrees of variability under both control and high-light conditions. Except for proline content, most traits showed higher CVs under high-light stress (16.79~57.48%) compared with the control (13.82~42.86%). The pronounced variation under high-light conditions highlights the discriminative power of these traits, supporting their suitability as effective indicators for evaluating high-light tolerance in oat. However, responses to high-light stress varied among individual traits, and no single trait could comprehensively reflect genotypic tolerance. Therefore, an integrated multi-trait approach is necessary for a robust assessment of high-light tolerance.

### 3.3 Correlation analysis

**Figure 3:**
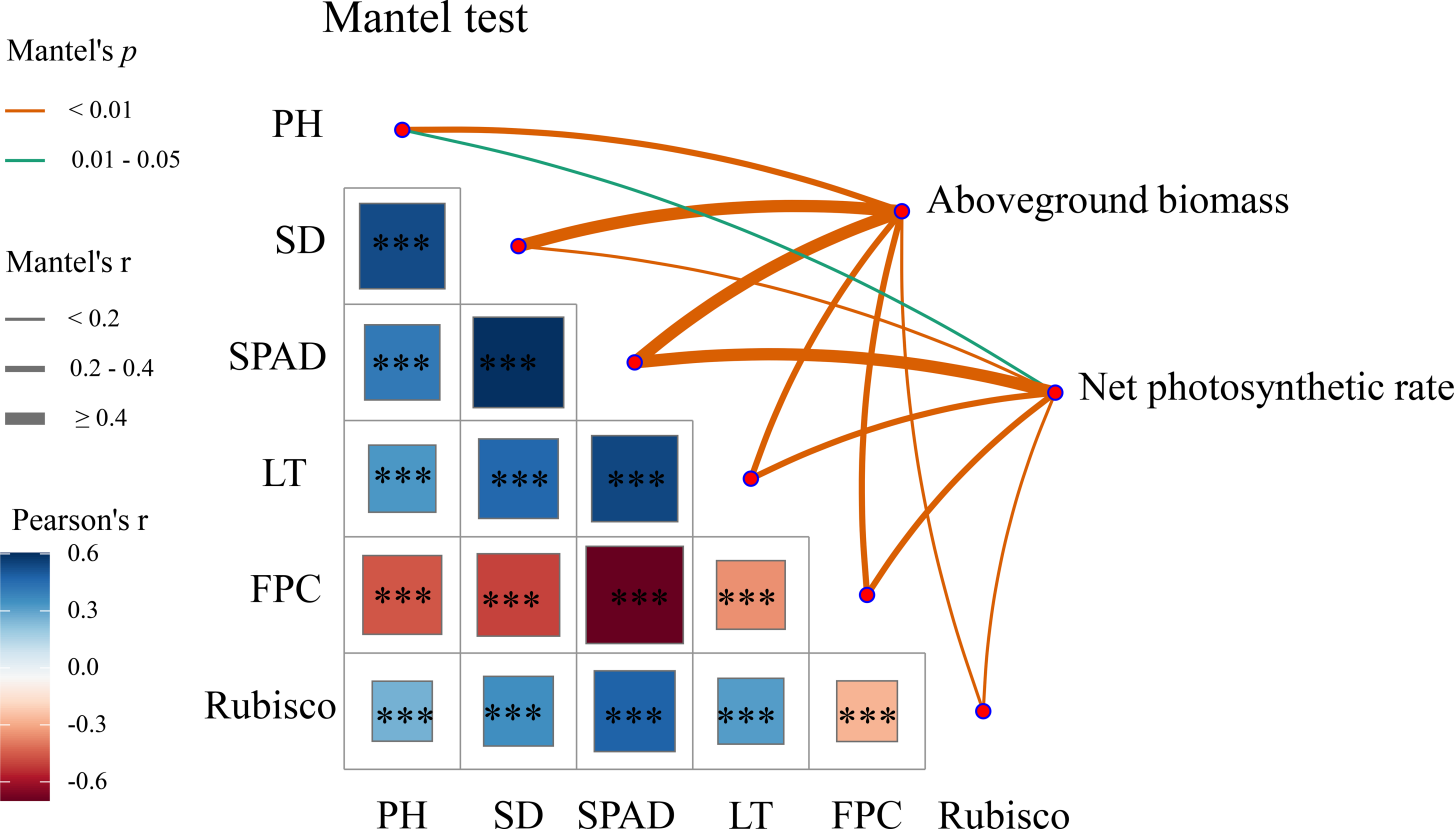
Correlation network among trait groups based on mantel test. The color gradient in the matrix indicates the Pearson correlation coefficient, with blue representing positive correlations and red representing negative correlations. Asterisks indicate significance levels, ***: *P* < 0.001. In the Mantel network, line thickness represents the strength of the Mantel correlation coefficient (r), where r < 0.2, 0.2 - 0.4, and ≥ 0.4 indicate weak, moderate, and strong correlations, respectively. Line color represents the significance level of the Mantel test (*p* value).

Pearson correlation analysis and Mantel tests were performed on seedling agronomic traits, net photosynthetic rate, proline content, Rubisco activity, and aboveground biomass (**Figure 3**). The Pearson analysis indicated that plant height, stem diameter, leaf thickness, and Rubisco activity were all strongly positively correlated with SPAD value (*P* < 0.001), whereas these traits exhibited highly significant negative correlations with proline content (*P* < 0.001). Mantel tests further revealed that aboveground biomass was strongly positively associated with stem diameter and SPAD value (Mantel’s r>0.5, *P* < 0.01), and also showed significant positive correlations with plant height, leaf thickness, proline content, and Rubisco activity (*P* < 0.01), highlighting the key roles of these traits in aboveground biomass accumulation. Additionally, net photosynthetic rate was strongly positively correlated with SPAD value (Mantel’s r>0.4, *P* < 0.01), and exhibited significant positive correlations with plant height, stem diameter, leaf thickness, proline content, and Rubisco activity (*P* < 0.05), suggesting a coordinated relationship between photosynthetic efficiency and both morphological and physiological traits in oat seedlings.

### 3.4 Integrated evaluation and cluster analysis

**Figure 4:**
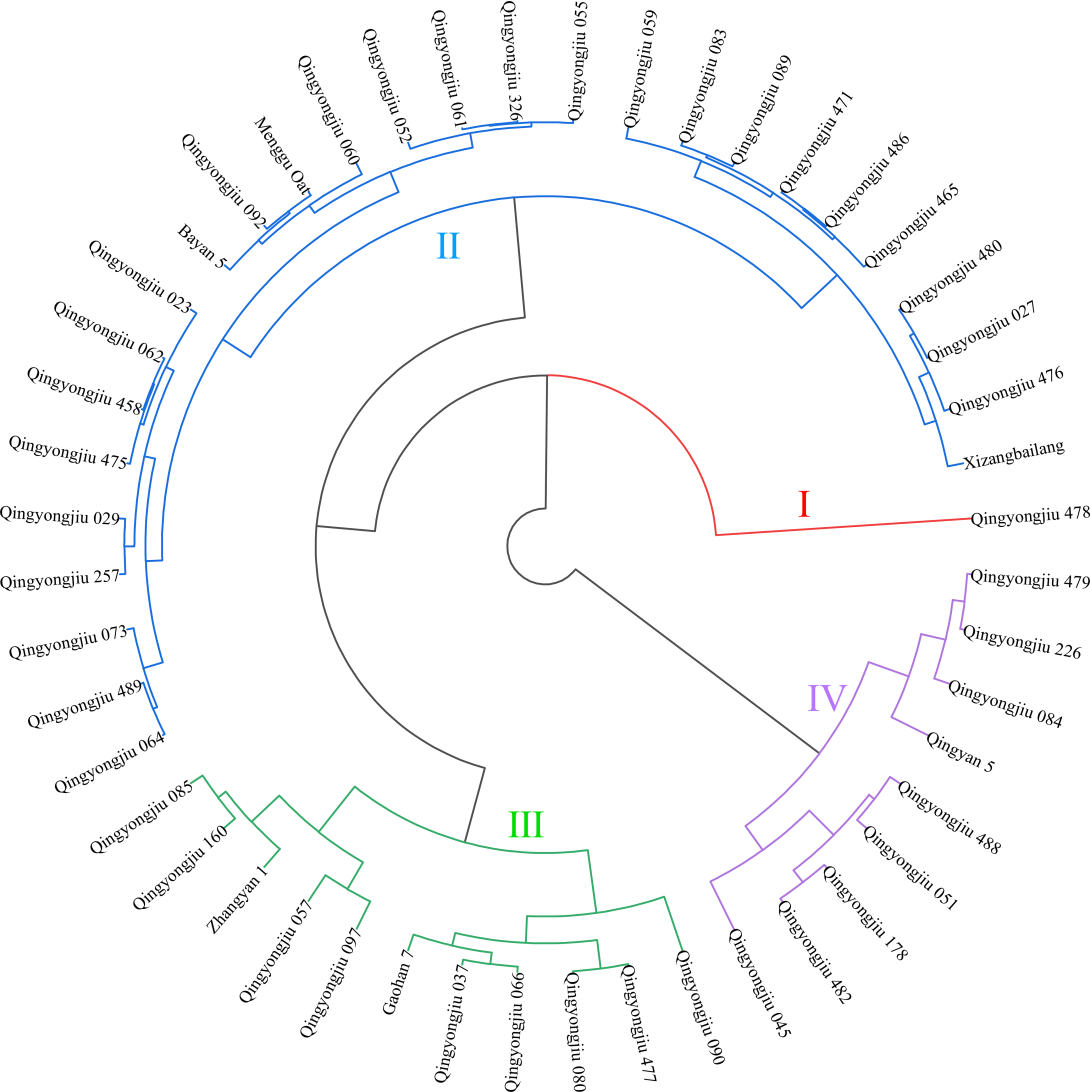
Cluster analysis of high-light tolerance in 48 oat germplasm resources.

To comprehensively evaluate the high-light tolerance of 48 oat genotypes, a TOPSIS multi-criteria decision-making model was employed to integrate the high-light stress responses of all measured traits in a weighted manner. The relative closeness (Closeness Coefficient, CI) of each genotype was calculated and used to rank the materials (**Table 3**). CI values ranged from 0.190 to 0.806, with higher values indicating stronger overall tolerance to high-light stress. Based on Euclidean distance clustering of the CI values (**Figure 4**), the 48 genotypes were classified into four tolerance categories: Level I, extremely tolerant (1 genotype); Level II, tolerant (27 genotypes); Level III, sensitive (11 genotypes); and Level IV, highly sensitive (9 genotypes). Clear differentiation was observed among these categories. Notably, Qingyongjiu 478 and Xizangbailang exhibited the highest CI values of 0.806 and 0.698, respectively, indicating strong adaptation potential under high-light conditions, whereas Qingyongjiu 226 and Qingyongjiu 479 had CI values below 0.2, reflecting relatively weak overall tolerance. These findings systematically capture the adaptive divergence of oat germplasm under high-light stress and provide a robust foundation for the precise identification and targeted breeding of high-light-tolerant genotypes.

**Table 3:** Comprehensive evaluation index values for the high-light response of 48 oat genotypes.

Code	Germplasm resources	CI values	Ranking results	Code	Germplasm resources	CI values	Ranking results
V1	Qingyongjiu 471	0.666	9	V25	Qingyongjiu 023	0.605	20
V2	Qingyongjiu 465	0.668	6	V26	Qingyongjiu 027	0.683	4
V3	Qingyongjiu 458	0.600	22	V27	Qingyongjiu 062	0.601	21
V4	Qingyongjiu 488	0.255	44	V28	Qingyongjiu 064	0.571	28
V5	Qingyongjiu 085	0.550	29	V29	Qingyongjiu 097	0.470	33
V6	Qingyongjiu 084	0.205	46	V30	Qingyongjiu 160	0.538	30
V7	Zhangyan 1	0.524	31	V31	Qingyongjiu 479	0.190	48
V8	Xizangbailang	0.698	2	V32	Qingyongjiu 480	0.683	5
V9	Qingyongjiu 045	0.315	40	V33	Qingyongjiu 486	0.667	7
V10	Qingyongjiu 051	0.265	43	V34	Qingyongjiu 482	0.277	41
V11	Qingyongjiu 080	0.404	37	V35	Qingyongjiu 092	0.615	18
V12	Qingyongjiu 083	0.661	10	V36	Qingyongjiu 090	0.369	39
V13	Qingyongjiu 257	0.585	25	V37	Qingyongjiu 061	0.636	14
V14	Qingyongjiu 326	0.637	13	V38	Qingyongjiu 060	0.625	16
V15	Qingyongjiu 476	0.687	3	V39	Qingyan 5	0.231	45
V16	Qingyongjiu 475	0.598	23	V40	Menggu Oat	0.619	17
V17	Qingyongjiu 477	0.403	38	V41	Bayan 5	0.611	19
V18	Qingyongjiu 478	0.806	1	V42	Gaohan 7	0.439	34
V19	Qingyongjiu 226	0.193	47	V43	Qingyongjiu 052	0.632	15
V20	Qingyongjiu 178	0.275	42	V44	Qingyongjiu 055	0.638	12
V21	Qingyongjiu 073	0.577	26	V45	Qingyongjiu 057	0.497	32
V22	Qingyongjiu 066	0.422	36	V46	Qingyongjiu 059	0.656	11
V23	Qingyongjiu 037	0.426	35	V47	Qingyongjiu 489	0.572	27
V24	Qingyongjiu 029	0.590	24	V48	Qingyongjiu 089	0.663	9

### 3.5 Key determinants and pathways influencing aboveground fresh weight

**Figure 5:**
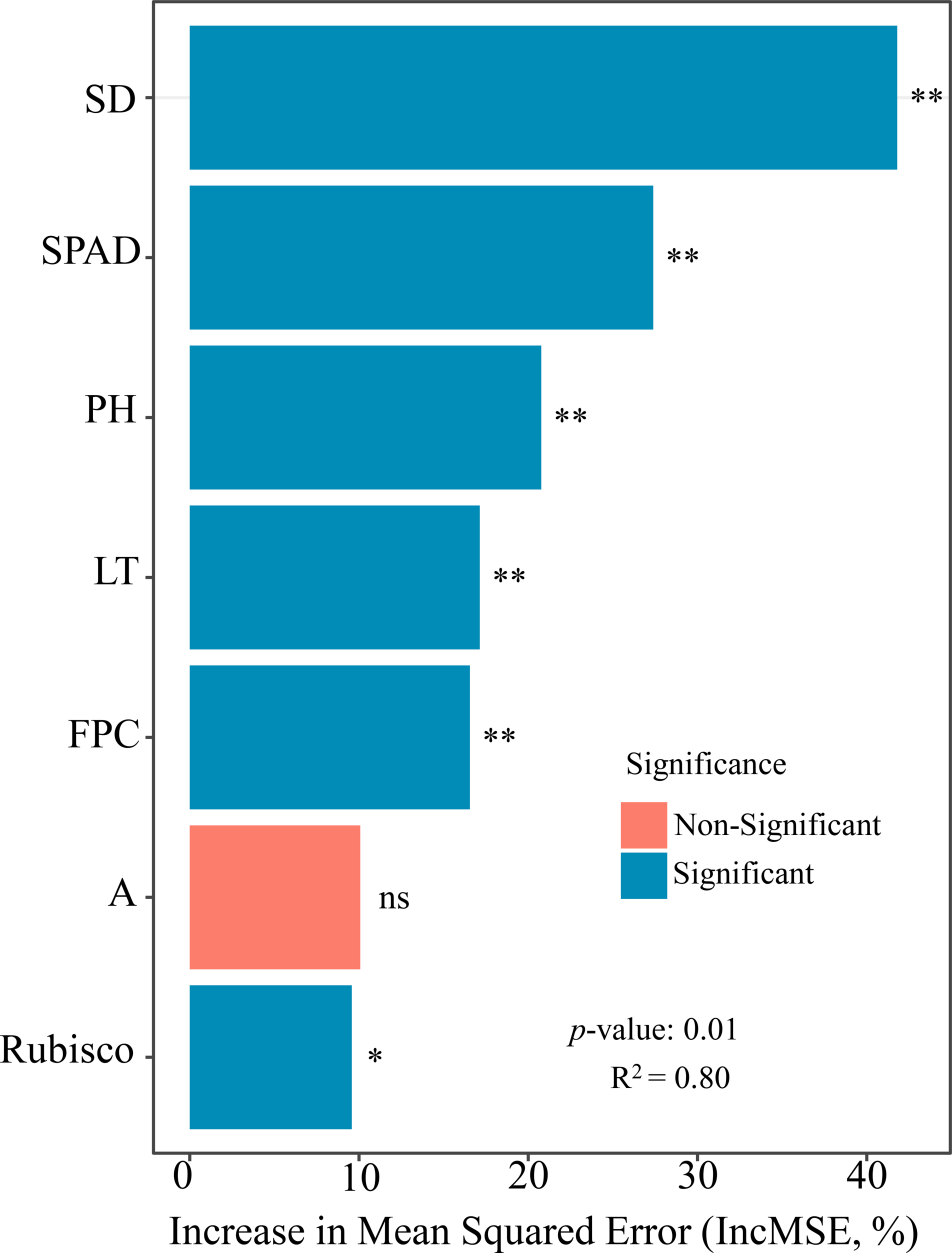
Variable importance ranking for predicting aboveground biomass based on a random forest model. The percentage increase in mean squared error (MSE) represents the importance of predictive variables. **P < 0.01.

To clarify the relative contributions of agronomic traits, photosynthetic characteristics, and proline accumulation to AFW in oat seedlings, a random forest model was applied to rank the importance of multiple traits (**Figure 5**), thereby identifying key determinants with high explanatory power. The analysis showed that stem diameter, SPAD value, plant height, leaf thickness, and proline content exerted highly significant effects on AFW (*P* < 0.01), whereas Rubisco activity had a significant but comparatively smaller effect (*P* < 0.05). These results indicate that AFW formation is jointly regulated by plant morphological traits, photosynthetic capacity, and osmotic adjustment processes.

**Figure 6:**
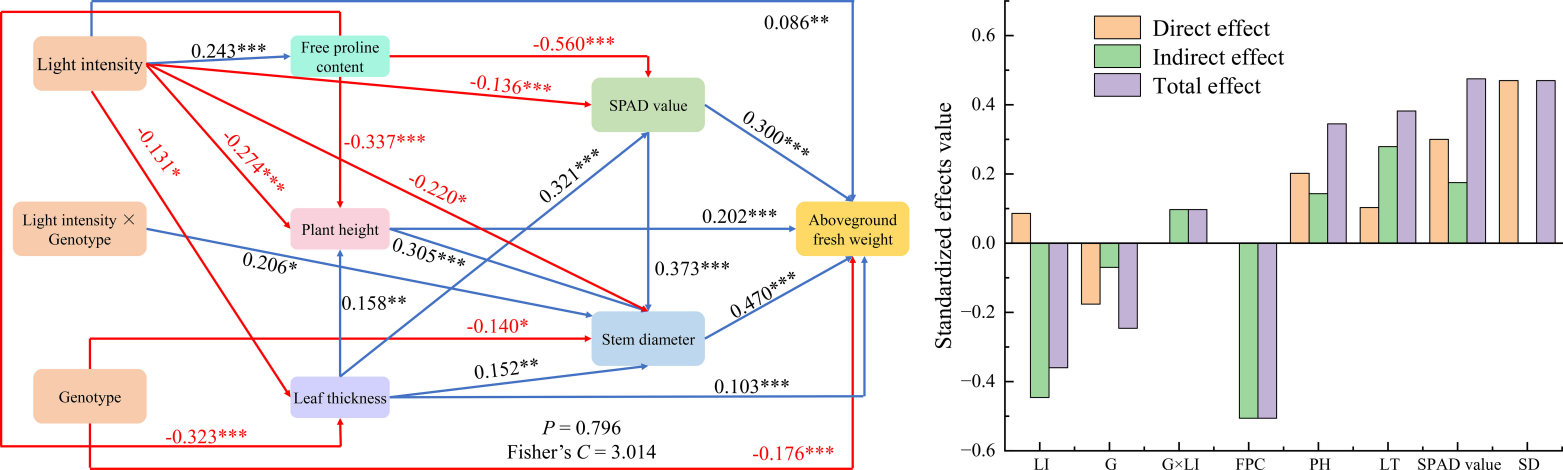
Structural equation model illustrating the pathways through which genotype and light intensity influence aboveground fresh weight, along with the standardized effect sizes of the associated variables. Blue arrows denote significant positive paths, red arrows significant negative paths. Values indicate standardized path coefficients. *: *P* < 0.05, **: *P* < 0.01, ***: *P* < 0.001. LI, Light intensity; G, Genotype; LI × G, Light intensity × Genotype; FPC, Free proline content; PH, Plant height; LT, Leaf thickness; SD, Stem diameter; AFW, Aboveground fresh weight.

Based on the key traits identified by the random forest analysis, a SEM was further constructed to disentangle the direct and indirect pathways through which these factors influence AFW (**Figure 6**). The SEM exhibited a good overall fit (*P* = 0.796, Fisher’s *C* = 3.104), suggesting that the proposed model adequately captured the relationships among variables. Path analysis revealed that genotype, light intensity, plant height, leaf thickness, SPAD value, and stem diameter all had significant direct effects on AFW, with standardized path coefficients of -0.176, 0.086, 0.202, 0.103, 0.300, and 0.470, respectively. In addition, genotype, light intensity, and their interaction indirectly affected AFW by modulating intermediate traits, including plant height, leaf thickness, SPAD value, and stem diameter. When both direct and indirect effects were integrated, proline content, SPAD value, and stem diameter exhibited the largest total effects on AFW (−0.506, 0.475, and 0.470, respectively), underscoring their pivotal roles in AFW formation at the seedling stage. Notably, proline content showed a pronounced negative effect on AFW, which is likely associated with stress-induced proline accumulation under high-light conditions. Such accumulation is widely recognized as an indicator of osmotic adjustment and stress-responsive physiological regulation, and in the present study it was consistently accompanied by a reduction in aboveground fresh biomass.

## 4 Discussion

### 4.1 Rationality of light intensity selection for high-light adaptability assessment at the oat seedling stage

In high-light adaptability assessments, the selection of an appropriate stress light intensity is a decisive factor in determining whether phenotypic and physiological differences among oat genotypes can be effectively distinguished, and thus represents a fundamental prerequisite for obtaining robust and reliable evaluation outcomes. When light intensity is insufficient, plants are generally able to maintain relative photosynthetic stability through intrinsic regulatory adjustments and photoprotective mechanisms, thereby masking genotype-specific response differences. In contrast, excessively high light intensity may trigger widespread photoinhibition and growth suppression, causing seedlings of different genotypes to converge toward a uniformly stressed state. Under either extreme, inter-genotypic variation in stress responses is inadequately expressed, ultimately compromising the accuracy of high-light tolerance evaluation in oat seedlings. Based on a comparative analysis of multiple light intensity treatments, this study identified 1600 μmol·m^-2^·^-1^ as an optimal irradiance for screening high-light tolerance at the seedling stage. Under this light regime, plant height, aboveground biomass, SPAD values, and proline content exhibited significant deviations from the control across all tested genotypes (*P* < 0.05), indicating a strong capacity to discriminate among materials with differing tolerance levels. Importantly, from an ecological adaptation perspective, an irradiance of 1600 μmol·m^-2^·^-1^ closely approximates the light conditions experienced by plants at midday on clear summer days in alpine regions, thereby providing a realistic simulation of the high-light environments that oats may encounter under natural field conditions. Furthermore, comparable light intensity ranges have been widely adopted in high-light stress studies of other crop species, including wheat (Wang et al., 2014), maize (Zhang et al., 2015), and tomato (Jiang et al., 2020), underscoring the broad applicability and physiological relevance of this irradiance level. Such consistency across species supports its suitability for elucidating photosynthetic response patterns and for conducting comparative analyses of tolerance differences among genotypes. Collectively, 1600 μmol·m^-2^·^-1^ represents a physiologically meaningful light intensity for the evaluation of high-light adaptation in oat seedlings.

### 4.2 Evaluation of high-light adaptability in oat seedlings

Under the background of global climate warming, abiotic stresses such as high temperature, high irradiance, and drought frequently co-occur, exerting pronounced impacts on crop growth and yield formation (Robles-Zazueta et al., 2024). High light not only directly constrains photosynthetic processes but also amplifies the detrimental effects of heat and drought stress. Consequently, a systematic evaluation of phenotypic and physiological responses of oat seedlings under high-light conditions is essential for identifying high-light-tolerant germplasm and improving stress adaptability.

Under high irradiance, absorbed light energy often exceeds the capacity of carbon assimilation, leading to the accumulation of excess excitation energy within the photosynthetic apparatus. This imbalance induces photoinhibition and ultimately reduces photosynthetic efficiency (Falcioni et al., 2024). In the present study, high-light stress markedly affected both growth performance and photosynthetic physiology of oat seedlings, with pronounced genotypic variation observed across multiple traits. These results indicate substantial genetic diversity in high-light adaptability among oat germplasm resources. From a morphological and growth perspective, high-light stress significantly suppressed seedling growth. AFW, a key indicator reflecting seedling vigor and plant water status, declined significantly under high-light conditions, with an average reduction of approximately 22%. Moreover, the increased coefficient of variation for AFW suggests considerable differences in sensitivity to high-light stress among genotypes. The reduction in fresh weight is likely associated with enhanced transpirational water loss and impairment of photosynthetic function under strong irradiance (Zhang et al., 2016), which together restrict assimilate accumulation. In contrast, aboveground dry weight did not show a significant change, suggesting that short-term high-light stress had a relatively limited effect on dry matter accumulation under the conditions of this experiment, and that the reduction in fresh weight was mainly related to changes in plant water status. However, under prolonged high-light exposure, persistent constraints on photosynthetic carbon assimilation may eventually influence biomass accumulation. Moreover, genotypic differences in water-use efficiency (WUE) may also contribute to the differential responses observed among germplasm resources, as variations in stomatal regulation and transpiration control can affect plant water balance and growth under high irradiance. Consistent with these responses, morphological traits including plant height, stem diameter, and leaf thickness exhibited overall decreasing trends under high-light stress. This pattern suggests that strong irradiance not only constrains biomass accumulation but also negatively affects morphological development in oat seedlings. Such responses may be associated with restricted cell elongation, limited assimilate availability (Balcke et al., 2024), and disruption of growth-related signaling pathways (Zhang et al., 2020). At the level of leaf photosynthetic physiology, high-light stress imposed multilevel constraints on light capture (Wobbe et al., 2016), photosynthetic efficiency, and downstream carbon assimilation processes. Leaf SPAD values declined overall under high irradiance, accompanied by a narrowing of variation, indicating impaired chlorophyll synthesis and stability and, consequently, reduced efficiency of light absorption and energy transfer. Correspondingly, net photosynthetic rate decreased significantly, with an increased coefficient of variation, suggesting that high-light stress broadly reduced photosynthetic capacity at the population level while revealing substantial genotypic differentiation in photosynthetic regulation and high-light tolerance. At the biochemical level, Rubisco, the key rate-limiting enzyme responsible for CO_2_ fixation, exhibited a significant overall decline in activity under high-light stress (Iñiguez et al., 2020). This reduction indicates that high irradiance constrains the catalytic capacity of carbon assimilation enzymes, thereby limiting CO_2_ fixation efficiency and restricting photosynthate production at the biochemical stage. In terms of osmotic regulation, high-light stress significantly promoted proline accumulation (Liang et al., 2013). As a well-established osmoprotectant, proline plays important roles in maintaining cellular osmotic balance, stabilizing proteins and membrane structures, and scavenging reactive oxygen species (Wang et al., 2024). The elevated proline levels observed in this study indicate the activation of stress-defense and adaptive regulatory mechanisms aimed at mitigating cellular damage induced by high-light stress. Taken together with the reductions in chlorophyll content, net photosynthetic rate, and Rubisco activity, these findings suggest that oat seedlings maintain cellular homeostasis under high-light stress by enhancing osmotic adjustment and defensive metabolic responses, reflecting coordinated regulation between photosynthetic carbon assimilation and stress defense. Overall, oat seedlings exhibited coordinated, multilevel responses to high-light stress encompassing growth performance, photosynthetic regulation, and osmotic defense, highlighting diverse adaptive strategies among different germplasm resources. Nevertheless, seedling biomass is jointly determined by multiple interacting traits, and the relative contributions and interactions among morphological, photosynthetic, and stress-related physiological traits require further quantitative analysis to elucidate their synergistic effects on growth performance under high-light conditions. Growth and physiological responses observed at the seedling stage may, to some extent, indicate the potential adaptability of different germplasm to high-light environments. Previous studies have reported associations between seedling stress responses and subsequent growth or yield traits (Li X. et al., 2025; Luo et al., 2024), suggesting that seedling phenotypic evaluation can provide a useful basis for the preliminary identification of high-light-tolerant germplasm. However, the extent to which seedling performance predicts adult plant growth and final yield remains to be verified through whole-season field investigations.

### 4.3 Contribution of key traits to aboveground fresh weight and elucidation of their pathways of action

By integrating random forest analysis with structural equation modeling, this study not only identified the key traits driving variation in AFW of oat seedlings but also elucidated the direct and indirect pathways through which these traits regulate biomass accumulation. This integrative framework reveals a hierarchical and interconnected regulatory network in which morphological traits, photosynthetic capacity, and osmotic adjustment jointly shape seedling growth under high-light conditions. Stem diameter, plant height, and leaf thickness exhibited significant positive direct effects on AFW, indicating that structural growth constitutes a fundamental determinant of aboveground biomass formation. Among these traits, stem diameter played a particularly prominent role, likely reflecting its dual function in mechanically supporting canopy expansion and enhancing vascular transport capacity for water and nutrients, thereby directly facilitating fresh biomass accumulation. This finding further substantiates the decisive role of morphological architecture in biomass production of gramineous crops (Liu Q. et al., 2025). At the functional trait level, the positive contribution of SPAD values highlights the central importance of photosynthetic pigment content in sustaining carbon assimilation (Colla et al., 2012). Enhanced SPAD values not only directly promoted AFW accumulation by improving photosynthetic efficiency but also indirectly influenced biomass formation through coordinated regulation of intermediate morphological traits such as plant height and leaf thickness, underscoring the close coupling between photosynthetic function and structural development. In contrast, proline content exerted a significant negative effect on AFW, indicating a trade-off between stress adaptation and biomass accumulation. Elevated proline levels under high-light stress likely reflect a reallocation of carbon and nitrogen resources toward osmoprotective and defense-related metabolism. Proline biosynthesis is an energy- and reductant-demanding process that requires substantial inputs of carbon skeletons, nitrogen, and reducing power (e.g., NADPH). Increased metabolic flux toward proline synthesis may therefore compete with primary metabolic pathways involved in photosynthate utilization and structural growth (Liang et al., 2013). As a result, excessive proline accumulation under stress conditions may limit biomass production by diverting metabolic resources away from growth-related processes. This pattern is consistent with previous reports describing stress-induced changes in resource allocation strategies in gramineous crops under abiotic stress conditions (An et al., 2024; Ganie et al., 2022), suggesting that proline accumulation may function not only as a protective osmolyte but also as a physiological indicator of stress intensity and growth limitation. Overall, these findings demonstrate that accurate evaluation of oat seedling performance under high-light stress requires an integrative consideration of structural traits, photosynthetic capacity, and stress-adaptive indicators. Reliance on single traits may obscure key regulatory interactions and underestimate the complexity of biomass formation mechanisms under adverse light environments.

### 4.4 Integrated assessment and identification of superior oat germplasm

High-light adaptability in oat at the seedling stage is fundamentally manifested as an integrated response of multiple morphological, physiological, and metabolic traits, arising from the coordinated regulation of growth establishment, photosynthetic performance, and stress-defense processes. Such integrated assessment approaches have been widely applied in the evaluation of abiotic stress tolerance in crops including wheat (Gupta et al., 2020) and maize (Liu et al., 2023). In this study, nine representative indicators covering plant height, aboveground biomass, net photosynthetic rate, proline content, and related traits were jointly integrated to reflect variation in growth performance, photosynthetic physiology, and osmotic regulation. The TOPSIS multi-criteria model was employed to calculate the relative closeness index, enabling a unified quantitative assessment of high-light tolerance across 48 oat germplasm accessions and providing an integrated depiction of their overall adaptive performance under strong light conditions. Based on the CI values, cluster analysis was further conducted to classify the oat germplasm into distinct high-light tolerance groups (Li L. et al., 2025). This classification not only allows for an intuitive visualization of tolerance gradients among genotypes but also facilitates the identification of germplasm exhibiting superior comprehensive adaptability. By jointly integrating the TOPSIS ranking and clustering results, Qingyongjiu 478 and Xizangbailang were identified as highly tolerant under high-light stress, characterized by coordinated maintenance of growth capacity, enhanced stability of photosynthetic function, and effective stress-regulation traits. Overall, the integrated evaluation framework established in this study provides an objective and robust characterization of genotypic variation in high-light tolerance among oat germplasm, offering a reliable methodological basis for the screening, utilization, and breeding of high-light adaptability oat resources.

## 5 Conclusion

In this study, variance analysis, cluster analysis, the TOPSIS multi-criteria decision model, and structural equation modeling were jointly employed to systematically assess the adaptability of 48 oat germplasm accessions under high-light stress. The results indicate that a light intensity of 1600 μmol·m^-2^·^-1^ constitutes an appropriate stress level for identifying high-light tolerance at the oat seedling stage. Moreover, proline content, SPAD value, and stem diameter were identified as dominant determinants of aboveground fresh weight formation, thereby representing key traits underlying high-light adaptability in oat seedlings. More importantly, this study establishes an integrated evaluation framework combining physiological traits, multi-criteria decision analysis, and causal modeling for the quantitative assessment of high-light adaptability in oat germplasm. High-light stress significantly suppressed seedling growth, with multiple growth-related traits exhibiting consistent declines. Based on a multi-trait comprehensive evaluation, two accessions, Qingyongjiu 478 and Xizangbailang, were identified as possessing superior adaptability under high-light conditions. These findings provide valuable germplasm resources and key physiological indicators for future studies on the physiological regulation, molecular mechanisms, and functional gene identification associated with high-light adaptability in oats.

## Data Availability

The original contributions presented in the study are included in the article/supplementary material. Further inquiries can be directed to the corresponding author.
